# *Streptococcus pyogenes* SpyCEP Influences Host-Pathogen Interactions during Infection in a Murine Air Pouch Model

**DOI:** 10.1371/journal.pone.0040411

**Published:** 2012-07-27

**Authors:** Nico Chiappini, Anja Seubert, John L. Telford, Guido Grandi, Davide Serruto, Immaculada Margarit, Robert Janulczyk

**Affiliations:** Research Centre, Novartis Vaccines and Diagnostics, Siena, Italy; The Methodist Hospital Research Institute, United States of America

## Abstract

*Streptococcus pyogenes* is a major human pathogen worldwide, responsible for both local and systemic infections. These bacteria express the subtilisin-like protease SpyCEP which cleaves human IL-8 and related chemokines. We show that localization of SpyCEP is growth-phase and strain dependent. Significant shedding was observed only in a strain naturally overexpressing SpyCEP, and shedding was not dependent on SpyCEP autoproteolytic activity. Surface-bound SpyCEP in two different strains was capable of cleaving IL-8. To investigate SpyCEP action *in vivo*, we adapted the mouse air pouch model of infection for parallel quantification of bacterial growth, host immune cell recruitment and chemokine levels *in situ*. In response to infection, the predominant cells recruited were neutrophils, monocytes and eosinophils. Concomitantly, the chemokines KC, LIX, and MIP-2 *in situ* were drastically increased in mice infected with the SpyCEP knockout strain, and growth of this mutant strain was reduced compared to the wild type. SpyCEP has been described as a potential vaccine candidate against *S. pyogenes*, and we showed that surface-associated SpyCEP was recognized by specific antibodies. *In vitro*, such antibodies also counteracted the inhibitory effects of SpyCEP on chemokine mediated PMN recruitment. Thus, α-SpyCEP antibodies may benefit the host both directly by enabling opsonophagocytosis, and indirectly, by neutralizing an important virulence factor. The animal model we employed shows promise for broad application in the study of bacterial pathogenesis.

## Introduction

The Gram-positive bacterium *Streptococcus pyogenes* is a strictly human pathogen. It causes a wide range of local and systemic infections, ranging from relatively common and trivial diseases such as tonsillitis and erysipelas, to life-threatening conditions such as streptococcal toxic shock syndrome, septicemia, and necrotizing fasciitis. The estimated global burden of disease due to *S. pyogenes* pharyngitis is 616 million cases annually. The corresponding estimate of invasive infections is 663,000, causing 163,000 deaths each year [1]. SpyCEP (*S. pyogenes* cell envelope protease, also called ScpC) was originally described in a clinical isolate from a case of necrotizing fasciitis, where broth culture supernatants contained interleukin 8 (IL-8)-degrading activity [2]. Subsequent partial purification of the protease and genetic manipulation of *S. pyogenes* strains resulted in the identification of a subtilisin-like protease responsible for the IL-8 cleavage [3], [4]. IL-8 is an important chemokine participating in the chemotactic recruitment of polymorphonuclear cells (PMN) to a site of injury or infection [5]. SpyCEP is highly conserved and paralogous to the C5a peptidase ScpA [4], a well-characterized virulence factor that interferes with host defences by cleaving and inactivating the chemotactic C5a peptide [6]. Analogously, IL-8 treated with culture supernatants from SpyCEP-expressing *S. pyogenes* showed a reduced capacity to induce PMN transmigration *in vitro* [3]. In soft-tissue infection models, histopathology showed increased PMN infiltration following infection with SpyCEP mutant strains when compared to the wild type (w.t.) strain [4], [7]. SpyCEP mutant strains also had impaired virulence in soft-tissue, subcutaneous, and intranasal (i.n.) infection models [4], [8], [9], while increased virulence was seen for a SpyCEP mutant strain in a sepsis model [7], and in one case with a soft tissue infection model [10]. Expression of SpyCEP is negatively regulated by the two-component signal transduction system CovRS and, in some strains, by another two-component system (Sil) and the cognate peptide pheromone [4], [10], [11]. Hypervirulent clinical isolates with CovRS mutations show overexpression of SpyCEP [11].

The domain architecture of SpyCEP comprises a subtilisin-like protease domain in the N-terminal part of the protein, and a C-terminal region of unknown function followed by a canonical LPXTG cell wall attachment motif. Recombinant expression of SpyCEP fragments showed that the protease domain alone is not sufficient for cleavage of IL-8 *in vitro*, but that at least the proximal part of the C-terminal needs to be present [12], [13]. We recently showed that the protein exhibits atypical auto processing and that one of the residues in the catalytic triad is located in an N-terminal fragment of the protein, which is released upon autocatalytic hydrolysis. Interestingly, the fragment remains non-covalently associated with the rest of the protein, and thus preserves proteolytic activity [14]. *In vitro* cleavage assays with recombinant SpyCEP (rSpyCEP) showed that not only IL-8 but several other chemokines are cleaved [8], [10], [12], [14]. SpyCEP is also considered a vaccine candidate following its identification by independent high throughput screening strategies [15]–[17], although the mechanism of protection is incompletely understood. In this study we asked a series of related questions regarding the role of SpyCEP in virulence and as a vaccine constituent. Is SpyCEP secreted/shed or surface anchored? Can surface-attached SpyCEP cleave IL-8? What are the specific contributions of SpyCEP to virulence and host response *in vivo*? Answers to these questions, together with an understanding of potential antibody interference with SpyCEP action, will provide a better understanding of the potential role of SpyCEP in a vaccine against *S. pyogenes*.

## Materials and Methods

### Ethics Statement

Animal treatments were performed in compliance with Italian laws, and approved by the institutional review board (Animal Ethical Committee, AEC nr 200819) of Novartis Vaccines and Diagnostics, Siena, Italy. The procedures were authorized by the Italian Ministry of Health (decree 111/2008-B).

### Bacterial strains and mutagenesis

*S. pyogenes* M1 strains 3348 (Istituto Superiore di Sanità, Rome, Italy) and SF370 (University of Siena, Italy) were grown in Todd-Hewitt broth supplemented with 0.5% yeast extract (THY medium) or on THY agar plates with 5% sheep blood, at 37°C in 5% CO_2_ atmosphere. The growth of *S. pyogenes* was monitored by optical density at 600 nm (OD_600_) using an Ultrospec 10 cell density meter (Amersham Biosciences). *Escherichia coli* DH5α, and BL21(DE3) (Invitrogen) were used for cloning, plasmid propagation and protein purification. *E. coli* were grown at 37°C in Luria-Bertani (LB) liquid medium with agitation or on LB agar plates. Antibiotics were added to the medium at the following final concentrations: 0.5 µg/ml or 1 µg/ml erythromycin (Erm) with *S. pyogenes*, and 100 µg/ml ampicillin with *E. coli*. The recombinant proteins rSpyCEP and rSpyCEP* were purified as previously described [14]. The in-frame deletion mutant 3348Δ*spyCEP* was previously described [14]. The SF370Δ*spyCEP* mutant was obtained in a similar way, using the same construct pJRS233::Δ*spyCEP*. To obtain strains 3348spyCEP* and SF370spyCEP* by gene replacement mutagenesis, the previously obtained construct pET21b+*spyCEP*(D151A) [14] was used as a template for PCR using primers spyCEP*F1 (TCGGATCCGGAAGCGTTTTCTTGGTGATG) and spyCEP*R1 (CCCTCGAGTGGAG TCCCTTCTTTAGGGG). The amplicon obtained was cloned into pJRS233 [18] using *Bam*HI and *Xho*I restriction sites, generating the new construct pJRS233spyCEP*. *S. pyogenes* were transformed by electroporation, and plasmid insertion/duplication and excision were performed essentially as described [18], [19]. Transformants were selected by growth on THY(Erm) plates at 30°C, and integration events were selected at the non-permissive temperature 37°C, and verified by PCR. After 5 passages permitting allelic exchange by homologous recombination and excision of the plasmid, Erm^S^ colonies were selected by replica plating. PCR sequencing on gDNA from the selected clones confirmed the base substitution 452A>C in spyCEP* and excluded secondary mutations in the locus.

### Preparation and analysis of bacterial extracts

Supernatant and cell wall proteins were obtained from 10 ml cultures grown until exponential and stationary phases. For cell wall extracts, bacterial pellets were washed in PBS, resuspended in 200 µl of 100 mM KPO_4_ (pH 6.1), 40% (w/v) sucrose, 200 U mutanolysin (Sigma), 1x Complete Mini EDTA-free protease inhibitor cocktail (Roche), and incubated at 37°C for 1 h. Proteins in the culture supernatant were precipitated by trichloroacetic acid at a final concentration of 10% (v/v) and incubation at 4°C for 16 h. The precipitate was washed in acetone and resuspended in 200 µl PBS. Proteins were separated by sodium dodecyl sulfate–polyacrylamide gel electrophoresis (SDS-PAGE) using MES 12% Bis-Tris gels (Invitrogen) and transferred to nitrocellulose membranes with the iBlot® system (Invitrogen). Immunodetection was performed using 1∶30,000 (3348 w.t. and mutant strains) or 1∶10,000 (SF370 w.t. and mutant strains) rabbit polyclonal α-rSpyCEP* serum, and 1∶20,000 goat α-rabbit IgG (H+L)-HRP conjugate (Bio-Rad). Blots were developed using SuperSignal West Pico Chemiluminescent Substrate (Thermo Scientific).

### *In vitro* IL-8 cleavage

All the *in vitro* cleavage assays were comprised of 10 µg/ml of recombinant IL-8 (PeproTech®) in 50 µl PBS, including additional recombinant proteins, extracts or bacteria (see below). Cleavage reactions were performed for 2 hours at 37°C. Proteins were then separated by SDS-PAGE on 18% Tris-Glycine gels (Invitrogen). IL-8 was detected by silver staining with Silver Quest™ (Invitrogen). For spike experiments, 3348Δ*spyCEP* cell wall extracts were incubated with IL-8 and 0–5 ng of rSpyCEP, and compared with reactions containing 3348 cell wall extracts (with native SpyCEP) and IL-8. For IL-8 cleavage with live bacteria, 10 ml of w.t. or mutant cultures were grown to OD_600_ = 0.4 (exponential phase), washed, and concentrated 200 fold in PBS. Alternatively, bacteria were grown o/n, diluted to OD_600_ = 0.4 in PBS, and concentrated 200 fold (stationary phase). 40 µl of bacterial suspension (∼10^9^ CFU) were incubated with IL-8 and 10 µg/ml of chloramphenicol in a total reaction volume of 50 µl. Serial dilutions of live bacteria were performed in 4 fold steps starting with 10^7^ (strain 3348) or 10^8^ CFU (strain SF370). After incubation and centrifugation, supernatants were collected and analyzed by SDS-PAGE as above.

### SpyCEP surface detection by flow cytometry

Cell wall-attached SpyCEP in 3348, 3348Δ*spyCEP*, 3348spyCEP*, SF370, SF370Δ*spyCEP* and SF370spyCEP* was analyzed by flow cytometry. Bacteria grown to exponential phase were centrifuged at 3000× *g*, washed in PBS and resuspended in 250 µl newborn calf serum (NCS) for 20 minutes at 25°C with shaking. Bacteria were then incubated at 4°C for 1 hour with mouse α-Alum (mock immunization with Alum) or mouse α-SpyCEP sera diluted 1∶200 in PBS, BSA 0.1% (PBSA). Samples were washed twice in PBSA and labeled using goat α-mouse phycoreythrin (PE)-conjugate secondary antibodies (Jackson Immuno Research) diluted 1∶50 for 30 minutes at 4°C. Bacteria were fixed in 2% paraformaldehyde (PFA) and washed in PBS. Data were collected using a BD FACS CANTO II (BD Bioscience) by acquiring 10,000 events. Data analysis was performed with Flow-Jo software (v.8.6, TreeStar Inc.)

### PMN transwell migration

PMN from heparinized whole mouse blood were purified by adding 5 volumes of erythrocyte lysis buffer (150 mM NH_4_Cl, 10 mM KHCO_3_, 0.1 mM EDTA, pH = 7.3), incubated for 8 min on ice, and the reaction was terminated by adding 45 volumes of cold PBS. Neutrophils were then purified by magnetic assisted cell sorting using mouse α-Ly6G-PE (BD Pharmingen), α-PE microbeads and MACS LS columns (Miltenyi Biotec) according to the manufacturer's instructions. Human PMN were isolated as described [20].

Transmigration was performed on 3 µm pore transwell plates (Millipore), with 5×10^4^ PMN/well in the upper chamber. KC or IL-8 were diluted in RPMI-PSG, 10% FCS, ranging from 0–1 µg/ml (KC) and 0–30 ng/µl (IL-8) using 3 fold dilution steps. The proteins rSpyCEP or rSpyCEP* were added at a concentration of 50 ng/µl, and samples (including the positive control without protease) were incubated at 37°C for 2 h. Antibody functional inhibition of rSpyCEP was assessed by an initial preincubation of rSpyCEP with (1∶500) rabbit polyclonal α-SpyCEP or α-Spy0269 (control) antibodies for 20 minutes at room temperature. Subsequently, the treated IL-8/KC samples were transferred to the lower chamber wells and the plates were incubated for 90 min at 37°C. PMN in the lower chamber were fixed in 2% PFA for 30 min, and counted by flow cytometry using a LSR II SOS (Becton Dickinson).

### Air pouch experiments and analysis

Eleven week old female CD1 mice (Charles River Laboratories) were used for the animal experiments in accordance with institutional and Italian guidelines for animal research. Dorsolateral air pouches were inflated by subcutaneous injection of 3 ml air on day 1 and day 4. On day 6, 1×10^7^ CFU of exponential phase (OD_600_ = 0.4) *S. pyogenes* diluted in 1 ml PBS were injected into the pouch. Strain SF370 was also used at a higher dose (1×10^8^). In all experiments, the inocula were subjected to viable counts by plating. At 2, 4 or 24 h after infection, the animals were euthanized, and an air pouch lavage was performed by repeated injection/aspiration of 2 ml PBS. 200 µl of lavage material from the air pouches were frozen at −80°C to promote cell lysis and permit release of intracellular bacteria. Bacterial load was determined by viable counts of thawed samples.

#### Cell recruitment

the cellular fraction of the lavage was obtained by centrifugation for 7 min (320× *g*, 4°C). The supernatant was collected for subsequent analysis (see below). Cells were resuspended in 1 ml of PBS with 1∶1000 of Live/Dead-AquaFluor (Invitrogen) for 30 min at 4°C. Cells were then fixed in 2% PFA, centrifuged as above, and resuspended in 25 µl staining solution for 30 min at 4°C with a combination of the following cell marker antibodies: α-Ly6C-FITC, α-CD11b-PE-Cy7, α-Ly6G-PE, α-CD11c-APCeFluor780, α-Ly6C+α-Ly6G (GR1)-PER-CP (from BD Pharmingen) and α-F4/80-PacificBlue, α-MHC-II-A700 (from eBioscience). The stained cells were analyzed using a LSR II SOS (BD) and BD DIVA software (BD Bioscience). In particular, we identified neutrophils (Ly6G^high^, GR1^high^), eosinophils (Ly6G^int^, F4/80^int^, SSC^high^), monocytes (CD11b^high^, CD11c^−^, Ly6C^high^, GR1^+^), dendritic cells (CD11c^+^, MHC-II^+^), and macrophages (CD11b^+^, F4/80^high^).

#### Chemokine analysis

chemokine concentrations in the air pouch lavage were measured at 2, 4 and 24 h after infection. Lavage material from the air pouch was centrifuged at 320× *g*, 4°C for 7 min and supernatants were filtered through 0.22 µm filters to remove bacteria. Chemokine concentrations were measured by MILLIPLEX™ MAP kit (Millipore™) according to the manufacturer's instruction using a mouse 13-plex custom panel.

In a separate experiment, *in vitro* digestion of the 13 chemokines (standard provided with the kit) was performed by coincubation with 0.5 µg/ml of rSpyCEP or rSpyCEP* at 37°C for 4 and 24 h.

### Statistical analysis

Statistical analysis was performed with GraphPad Prism software for Windows (version 5.00; GraphPad Software). Data sets were first analyzed for normal distribution and variance in order to choose appropriate statistical tests.

## Results

### Localization and activity of SpyCEP varies with growth-phase

**Figure 1:**
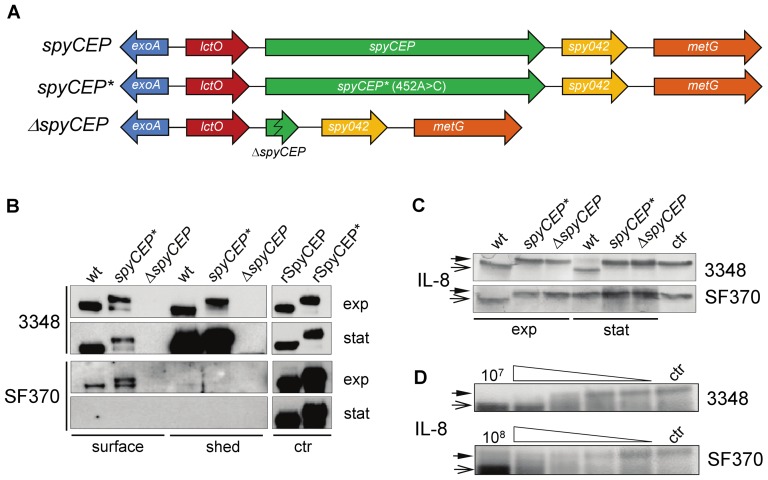
Localization and activity of SpyCEP. (**A**) Schematic representation of SF370 wild type and genetically manipulated *spy0416/spyCEP* loci. The same mutagenesis was conducted in the 3348 strain. The base substitution 452A>C is indicated (*). (**B**) Western blot analysis of cell wall extracts or supernatants from the 3348 and SF370 w.t. and mutant strains. SpyCEP was detected using rabbit polyclonal α-SpyCEP serum. 10 ng of rSpyCEP or rSpyCEP* were added as controls (ctr). (**C–D**) IL-8 cleavage assay with washed live bacteria. SDS-PAGE and silver staining were performed after digestion of IL-8 (10 µg/ml) in the presence of 10 µg/ml of chloramphenicol. Uncleaved and cleaved IL-8 are indicated by full and trace arrowhead respectively. Control lane (ctr) is IL-8 alone. (**C**) Strains were grown to exponential or stationary phase, resuspended in PBS at comparable bacterial densities (∼10^9^ CFU), and then incubated with IL-8. (**D**) Exponential phase bacteria 3348 w.t. (10^7^ CFU) and SF370 w.t. (10^8^ CFU) were serially diluted in PBS using 4 fold steps before incubation with IL-8.

Mutagenesis of *spyCEP* was performed in two M1 strains with diverse genetic backgrounds. 3348 is a strain with relatively high virulence in mice, and sequencing of the *covRS* locus showed the presence of several mutations inactivating the system (data not shown). Transcriptome analysis of invasive and non-invasive disease isolates has shown that several known virulence factors show a particular transcriptional profile associated with invasiveness, and that this is related to mutations in CovRS [21]–[23]. 3348 shows high expression of SpyCEP and SLO, together with a low expression of SpeB (data not shown), which is consistent with an invasive phenotype. SF370 was used to sequence the first complete genome of *S. pyogenes* [24], has an intact CovRS system, and is practically avirulent in traditional (i.p/i.n.) mouse challenge models (unpublished results). We obtained in-frame deletions of *spyCEP* in the SF370 strain, and the genotype was verified by sequencing of the locus. Independently, gene replacement was conducted, where the wild type (w.t.) *spyCEP* gene was replaced by spyCEP*. The latter encodes a mutated form of SpyCEP which is proteolytically inactive (D151A substitution) [14]. Sequencing of the spyCEP* locus verified that only the intended mutation was present after gene replacement. The w.t. and genetically manipulated loci are shown (Figure 1A). Growth curves of w.t. and isogenic mutant strains were compared and showed no discernible differences (Figure S1).

Surface extracts and bacterial growth supernatants from 3348Δ*spyCEP*, SF370Δ*spyCEP*, 3348spyCEP*, SF370spyCEP* and w.t. strains were analyzed by Western blot with α-SpyCEP antibodies (Figure 1B). Purified recombinant SpyCEP (rSpyCEP) and the inactive form rSpyCEP* were used as controls. Both the knockout strains showed the absence of a band corresponding to SpyCEP. In repeated experiments, an estimated 50–70% of total SpyCEP produced in strain 3348 was found associated with the bacterial surface in exponential phase. In contrast, by late stationary phase most (>90%) of the SpyCEP was found in the culture supernatant of strain 3348. Surface extracts from SF370 instead showed the presence of SpyCEP and SpyCEP* only in exponential phase. Significantly longer exposure and a threefold higher amount of primary antibodies were needed to visualize the proteins, suggesting an overall lower expression than in 3348. These data are consistent with a repression of *spyCEP* expression by the functional CovRS system in SF370. In the 3348spyCEP* and SF370spyCEP* strains we observed the unprocessed form of SpyCEP*, and a second band corresponding to the size of processed SpyCEP. Previous work has shown that SpyCEP* is incapable of undergoing autocatalytic processing *in vitro* [14]. The presence of a second band suggests that the cleavage site may be somewhat susceptible to cleavage by unknown protease(s) present in the extracts. Nevertheless, a protein thus processed should be proteolytically inactive due to the absence of a functional catalytic site (see below).

We hypothesized that surface-associated SpyCEP is active, and cleavage of IL-8 may occur independently of shed and/or secreted SpyCEP. W.t. and mutant bacteria were washed and incubated with IL-8. Complete cleavage of IL-8 by 3348 from exponential phase was observed, while there was no cleavage with 3348Δ*spyCEP* or 3348spyCEP* (Figure 1C). Similar results were obtained for the SF370 w.t. and mutants, suggesting that even a limited amount of surface-associated SpyCEP is sufficient for IL-8 cleavage. In stationary phase incomplete cleavage of IL-8 was seen for 3348, and no cleavage for SF370, consistent with the data on surface localization described above. To exclude the possibility that the activity observed depended on shed SpyCEP, the reactions were performed in the presence of chloramphenicol to block protein synthesis during the incubation. Moreover, in a control experiment Western blot analysis of precipitated supernatants from the reaction showed no detectable presence of SpyCEP (data not shown) - further strengthening that cleavage of IL-8 was by cell wall-attached SpyCEP only. By repeating the above experiment with serial dilutions of bacteria, the relative efficacy of SpyCEP-dependent cleavage in 3348 and SF370 was assessed. Compared to SF370, a 10 fold lower bacterial density of strain 3348 was sufficient to obtain a similar cleavage profile, suggesting a pronounced difference in SpyCEP expression/activity between the two strains (Figure 1D).

The streptococcal cysteine protease SpeB is a well-studied virulence factor that acts upon a variety of substrates and is regulated by CovRS [25], [26]. We examined SpeB levels in supernatants from bacteria in stationary phase, and noted dramatically higher levels of SpeB in SF370 compared to 3348 (data not shown). We hypothesized that the low quantity of SpyCEP in SF370 was related to SpeB activity. Consequently, bacteria were grown in the presence/absence of the specific cysteine protease inhibitor E64 (25 µM), and the localization experiment was repeated. However, extracts from bacteria grown in the presence/absence of E64 showed no differences in SpyCEP localization or amount (data not shown).

### A murine air pouch model to study the role of SpyCEP in *S. pyogenes* infection *in situ*

**Figure 2:**
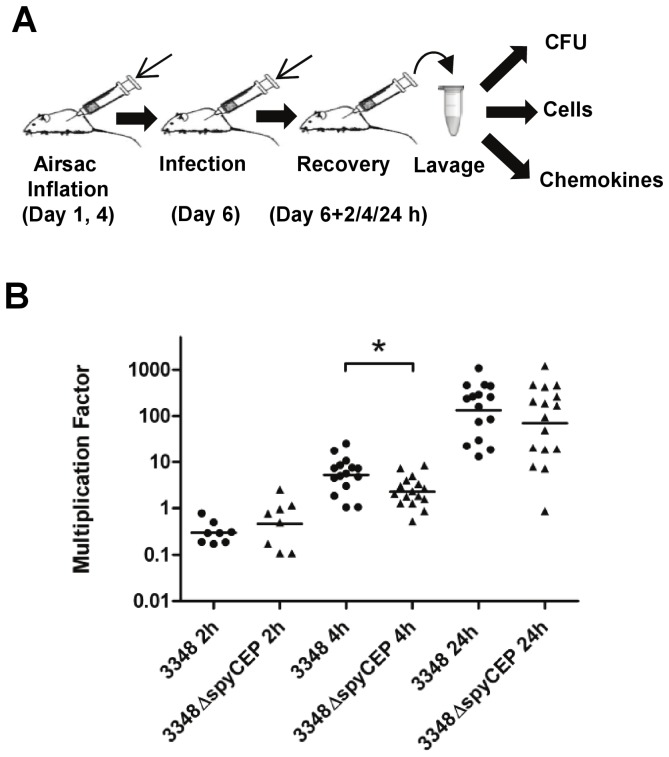
*S. Pyogenes* infection in a murine air pouch model. (**A**) Schematic of the *in vivo* air pouch model. For each experiment, 8 CD1 mice per group were infected with 1×10^7^ CFU of 3348, 3348Δ*spyCEP* or PBS. After air pouch inflation and bacterial infection, the lavage material was collected and fractionated for bacterial viable counts, leukocyte counts and chemokine analysis for each animal. (**B**) Bacterial multiplication factor (total CFU/inoculum CFU) in the lavage from individual mice. Time points 4 and 24 h represent aggregate data from two experiments. Horizontal bars are geometric means. Statistical significance (*) was tested by Mann-Whitney U, *P* = 0.014).

To investigate whether SpyCEP interferes with innate immune responses during *S. pyogenes* infection *in situ*, we adapted a murine air pouch model which has been extensively used to study inflammation [27], [28]. In brief, a dorsolateral air pouch was established by repeated injections of air. Bacteria were then injected into the air pouch, and 2, 4 or 24 h later a lavage with PBS was performed (Figure 2A). The lavage fluid was used for three types of analysis: the bacterial load was examined by viable counts, the presence and identity of immune cells were determined by flow cytometry, and chemokine levels were quantified by a multiplex bead ELISA. In initial experiments, dose-ranging was performed. We aimed to use the lowest possible dose that guaranteed bacterial survival and/or growth. In pilot experiments with 3348 and SF370 we established an infectious dose for 3348, while infection with SF370 resulted in >99% clearance of bacteria (Figure S2), and the strain was omitted from further study. Mice (n = 8 per group) were infected with 1×10^7^ CFU of w.t. 3348 or *spyCEP* knockout strains. Mock infection with PBS (n = 3 per group) was performed as a negative control, and viable counts on lavages (2, 4 and 24 h) showed no CFU. At 2 h post-infection, bacterial counts were similar to or lower than the starting inoculum (multiplication factor <1, Figure 2B). After 4 h, mice infected with 3348 and 3348Δ*spyCEP* had higher or equal bacterial loads *in situ* when compared to the starting inoculum. At 24 h, all mice except one showed considerable growth of bacteria (multiplication factor of 10–1000). The experiment was repeated for the two later time points, and aggregate data showed that 3348Δ*spyCEP* had a statistically significant decrease in CFU counts compared to the w.t. strain (>50% less bacteria in the mutant) at 4 h.

### *In situ* recruitment of leukocytes

**Figure 3:**
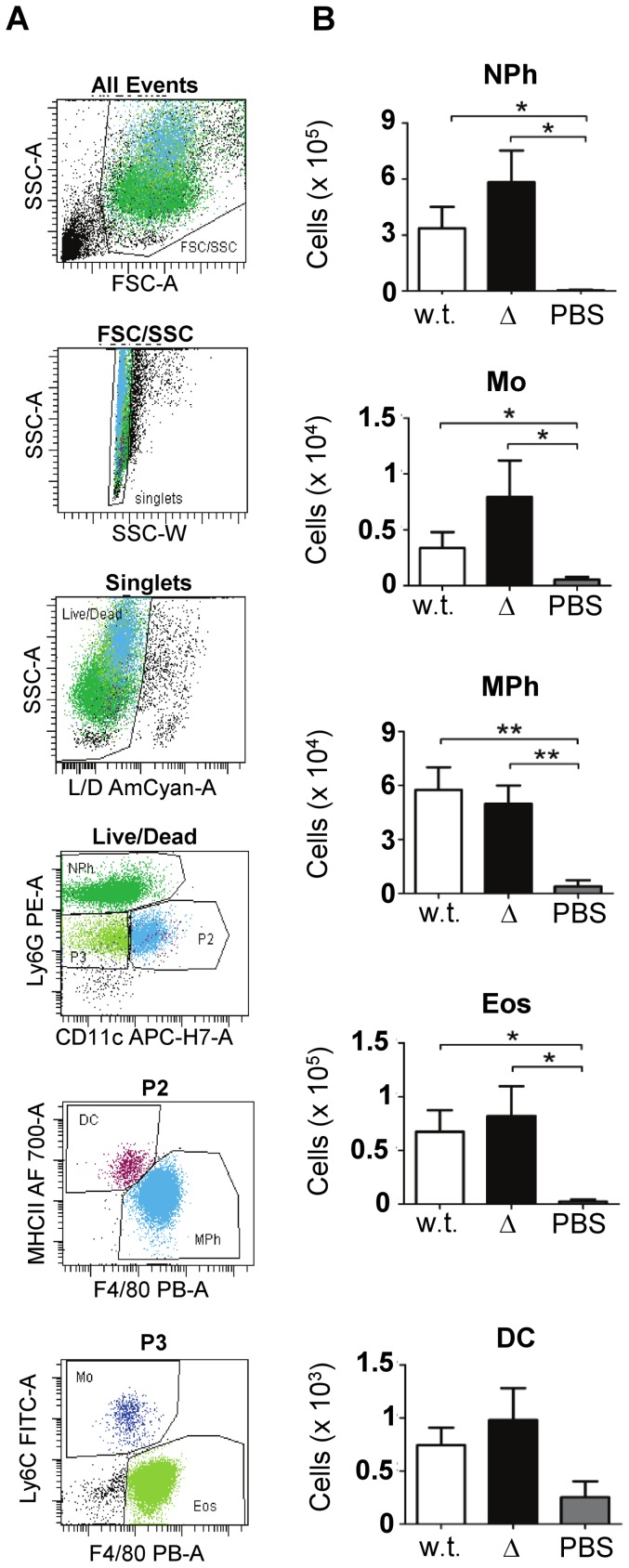
Cell recruitment *in situ*. (**A**) Gating strategy to identify neutrophils (NPh; Ly6G^high^, GR1^high^), dendritic cells (DC; CD11c^+^, MHC-II^+^), macrophages (MPh; CD11b^+^, F4/80^high^), monocytes (Mo; CD11b^high^, CD11c^−^, Ly6C^high^, GR1^+^) and eosinophils (Eos; Ly6G^int^, F4/80^int^, SSC^high^) 4 h post-infection. (**B**) Counts of each cell population identified in the lavage 4 h post-infection with 3348 (w.t.), 3348Δ*spyCEP* (Δ) or PBS. Data shown are means plus SEM of one representative experiment using 8 mice per group, except for the PBS control (n = 3). Statistical significance was tested by Mann-Whitney U: *P*<0.05 (*), *P*<0.01 (**).

Eukaryotic cells from the air pouch lavage of mice infected with 3348 and 3348Δ*spyCEP* were collected, stained for viability, fixed, and then stained with seven cell markers. The gating strategy involved successive identification of cells by morphology, vitality, and the presence/absence of specific cell markers. An overview of the cell types identified by combinations of markers is shown (Figure 3A). At 2 h, the number and type of live cells identified were similar to that in mock-infected mice, suggesting that cell recruitment in response to the infection was yet to occur (data not shown). At 4 h post-infection, the numbers of cell-like particles in the lavage from infected mice were in the range 10^5^–10^6^, and most cells were viable (78%). At 24 h post-infection, 10^6^ or more cell-like particles were identified, but only 0–1.3% represented live cells. While the total number of cell-like particles was higher (2–4 fold) at 24 h compared to 4 h, in terms of live cells there were 100 fold less. Concomitantly, the bacterial load *in situ* (see above) was up to 10 fold higher at 24 h than at 4 h. This suggests that the host cells failed to suppress the infection, and that the large number of cell-like particles mostly represent cellular debris. Consequently, conclusions about cell recruitment are only feasible at the 4 h time point. In absolute numbers, the predominant live cell population consisted of neutrophils, followed by eosinophils and macrophages (Figure 3B). Eosinophils are not known to have any role in the host response against *S. pyogenes* infection, and their presence in quantity was surprising. All cell types from the lavage except for dendritic cells were clearly elevated in infected mice compared to controls (mock infection with PBS). Mice infected with 3348Δ*spyCEP* showed a tendency to have more neutrophils and monocytes *in situ* compared to mice infected with the w.t. strain. This trend was consistent across three independent experiments. However, variability between individual samples was high and the trend was not statistically significant (Mann-Whitney U test, *P* = 0.1).

### SpyCEP specifically reduces chemokine levels *in situ* during infection

**Figure 4:**
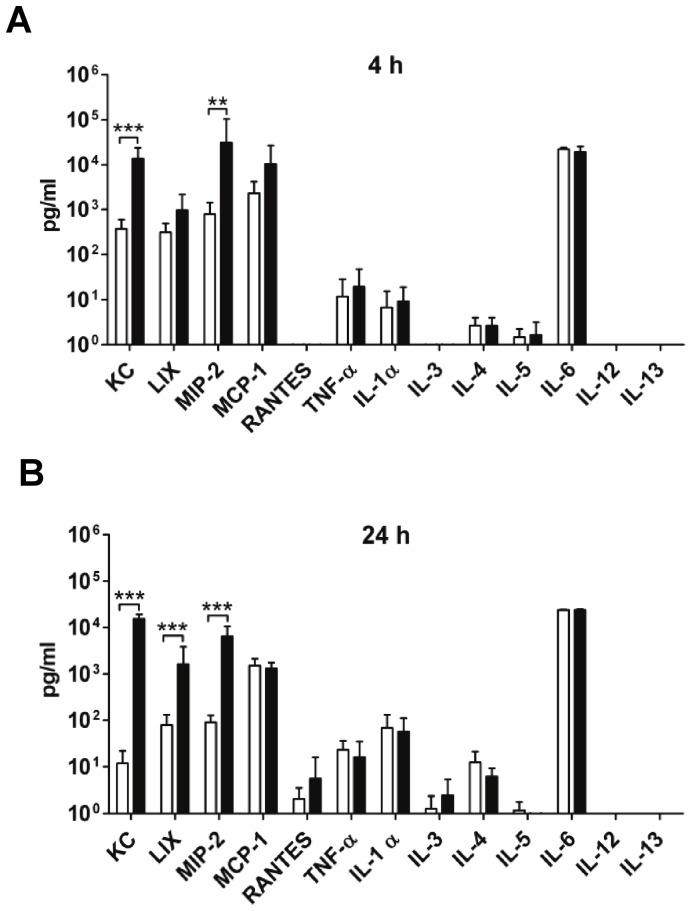
Increased levels of KC, LIX and MIP-2 during infection with 3348Δ*spyCEP*. Thirteen different chemokines were measured in the individual mouse air pouch lavages collected at 4 h (**A**) and 24 h (**B**) post-infection with 3348 (white bars) and 3348Δ*spyCEP* (black bars). Results are shown as means and SEM of one representative experiment using 8 mice per group. Statistical significance was tested by Mann-Whitney U: *P*<0.01 (**), *P*<0.001 (***).

In order to analyze the presence of chemokines *in situ*, 13 different chemokines in the air pouch lavage samples were measured (Figure 4). At 2 h post-infection, only KC (the murine functional homologue of IL-8) and IL-6 were detected, and KC levels in mice infected with 3348Δ*spyCEP* were significantly higher than in w.t. infected mice (data not shown). At 4 h post-infection, five chemokines (KC, LIX, MIP-2, MCP-1 and IL-6) were clearly elevated (Figure 4A) compared to samples from mock-infected animals, in which most chemokines were undetectable and in no case higher than 20 pg/ml. KC and MIP-2 were seen at concentrations above 10,000 pg/ml in the samples from mice infected with the 3348Δ*spyCEP* strain, while the samples from mice infected with the w.t. strain had at least 10 fold lower mean concentrations. The differences were statistically significant for both KC and MIP-2. At 24 h post-infection, the above chemokines were all still present at high concentrations (Figure 4B), and differences between w.t. and mutant were even more pronounced (10–1000 fold) and significant also for LIX.

In order to examine the specificity of SpyCEP against the chemokines used in our assay, the panel of 13 chemokines was also examined by incubation *in vitro* with rSpyCEP or rSpyCEP*. Subsequent measurement of chemokine levels showed that KC, LIX and MIP-2 levels decreased (100, 0 and 3 fold, respectively, at 4 h, and 200, 10 and 3 fold at 24 h) in samples incubated with rSpyCEP, while incubation with rSpyCEP* caused no such decrease (data not shown). The other 10 chemokines were unaffected by the treatment. As this experiment involves the simultaneous presence of competing substrates we conclude that among the 13 chemokines in the panel, only KC, LIX and MIP-2 are substrates for SpyCEP.

### Specific antibodies counteract rSpyCEP inhibition of PMN migration *in vitro*

**Figure 5:**
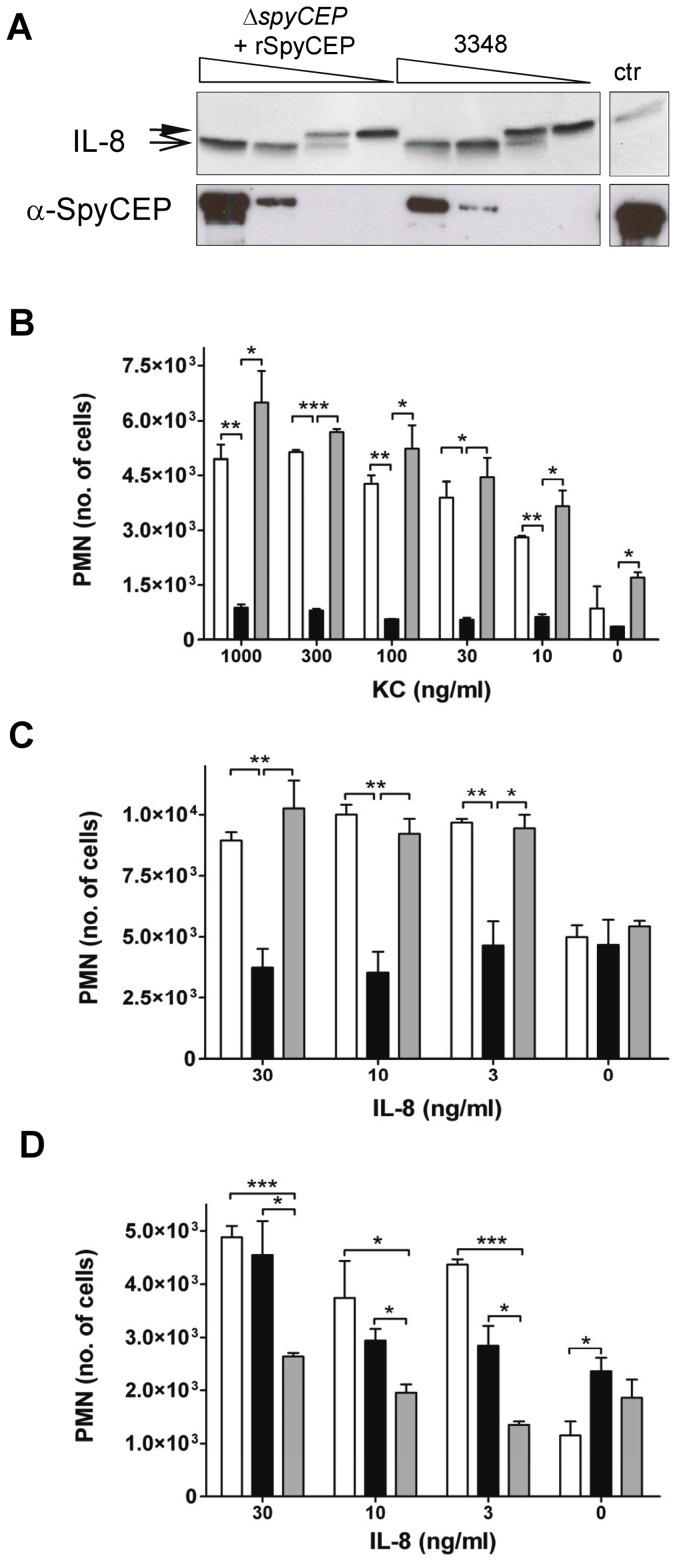
Activity of rSpyCEP and PMN transmigration. (**A**) Comparison of rSpyCEP and native SpyCEP activity on IL-8. SDS-PAGE (18% Tris-Glycine) and silver staining after 2 h of digestion with 5 ng, 1 ng, 0.2 ng, 0.04 ng or 0 ng (ctr lane) of rSpyCEP+3348Δ*spyCEP* extracts (left). Cell wall extracts containing comparable amounts of native SpyCEP from 3348 extracts (right). Full and trace arrowheads indicate intact and cleaved IL-8, respectively. Lower panel: control Western blot showing relative SpyCEP amounts compared to 5 ng of rSpyCEP (ctr). (**B,C**) SpyCEP effect on PMN transmigration. (**B**) Murine or (**C**) human PMN migration in response to KC or IL-8 (white), respectively, in the presence of rSpyCEP (black) or rSpyCEP* (grey). (**D**) Counteraction of rSpyCEP activity by specific antibodies. Human PMN migration using IL-8 (white); IL-8, rSpyCEP and α-SpyCEP (black); and IL-8, rSpyCEP, α-Spy0269 (grey). Data represent means plus SEM of one representative experiment using triplicates. Statistical significance was tested by unpaired Student T, (*) *P*<0.05, (**) *P*<0.01, (***), *P*<0.001.

In order to study PMN transmigration in a clean system, we first investigated whether rSpyCEP would constitute a suitable replacement for native SpyCEP on bacteria or in culture supernatant. IL-8 was incubated with serial dilutions of cell wall extract from the 3348 strain or from the isogenic mutant 3348Δ*spyCEP* where rSpyCEP had been added (Figure 5A). This ensured that potential inhibitory or activating constituents derived from the cell wall digestion procedure were equally present. The experiment covered a range of SpyCEP/rSpyCEP quantities, which induced non-detectable to complete IL-8 cleavage. The breakpoint (approximately equal proportions of cleaved and uncleaved IL-8) occurred at the same dilution for native SpyCEP and rSpyCEP, suggesting that the recombinant enzyme is fully active and can be used as a substitute for bacterial extracts or supernatant preparations.

Immunization with rSpyCEP induces protective immunity in murine models of *S. pyogenes* infection [15], [16], [29]. As SpyCEP is a virulence factor, the mechanism of protection may include opsonization and/or functional interference with SpyCEP activity. Previous studies have shown that IL-8 dependent transmigration of PMN *in vitro* is inhibited by SpyCEP from bacterial supernatants [3]. In a similar setup, we observed that pretreatment of KC and IL-8 with rSpyCEP reduces murine and human PMN recruitment, respectively. In the case of KC and murine PMN, a reduction of up to 8 fold was observed (Figure 5B), while for IL-8 the reduction was in the order of 2–3 fold (Figure 5C). Pretreatment with the inactive rSpyCEP* was used as a control and had no effect on human PMN transmigration, indicating that this functional interference is strictly dependent on proteolytic activity of rSpyCEP. In the case of murine PMN, rSpyCEP* showed a small increase in PMN migration over the whole KC concentration range compared to the control. We speculate that some impurities from the rSpyCEP* purification might result in this minor activation of murine PMN. In a short preincubation step, polyclonal antisera specific for rSpyCEP or for an unrelated *S. pyogenes* surface protein (Spy0269) were added to the system (Figure 5D). The control samples (with no IL-8) suggested that the addition of serum causes a slight increase in transmigration, perhaps due to presence of complement or chemokines. Nevertheless, the presence of α-rSpyCEP abrogated or alleviated the effect of SpyCEP on PMN transmigration, while α-Spy0269 antibodies had no effect. This suggests that immunization with rSpyCEP generates antibodies which can interfere functionally with the native *S. pyogenes* virulence factor.

**Figure 6:**
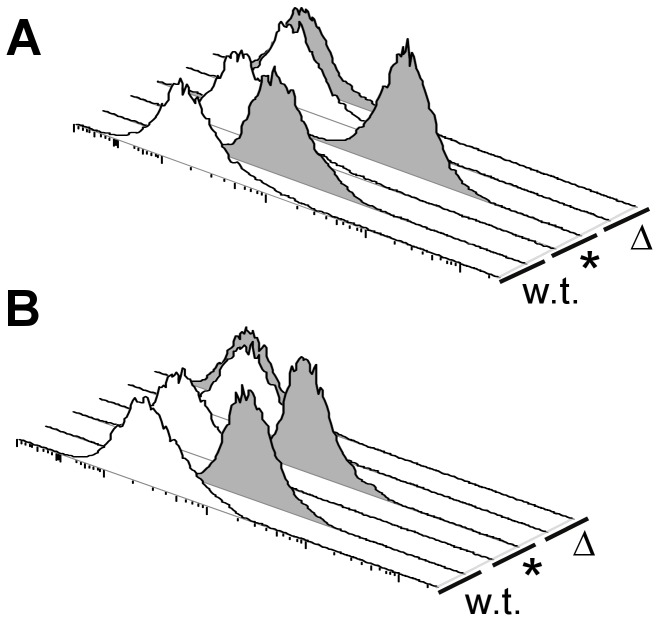
Specific antibodies recognize SpyCEP on bacteria. Exponential (**A**) and stationary (**B**) phase bacteria were labeled with pooled mouse α-Alum (white) or mouse α-SpyCEP (gray) sera. Secondary antibodies were rabbit α-mouse phycoerythrin conjugates, and the fluorescence (PE-A) is shown on the x axis. Strains 3348 (w.t.), 3348Δ*spyCEP* (Δ) and 3348spyCEP* (*) are displayed on the z axis.

SpyCEP, at least in exponential phase, was found attached to the bacterial surface, and active against IL-8 (see above). Moreover, the action of SpyCEP on chemokines and PMN recruitment was countered by the presence of specific antibodies. We next asked whether specific antibodies against rSpyCEP can recognize native SpyCEP on the bacterial surface. Bacteria in exponential and stationary phase were incubated with α-SpyCEP antisera, or sera from mice that were mock-immunized with Alum (neg. control). The 3348 w.t. and spyCEP* strains in exponential phase growth showed an appreciable shift in fluorescence intensity with α-SpyCEP antibodies compared to negative control samples (Figure 6). Knockout strains were similar to the negative control. In stationary phase, similar results were observed for strain 3348 and its mutants. With SF370, backgrounds were higher, and no appreciable shifts were observed compared to control samples (data not shown) in either phase of growth. In conclusion, recognition of SpyCEP at the bacterial surface requires a relatively high level of expression, such as in the overexpressing strain 3348.

## Discussion

Most of the studies on SpyCEP have focused on the secreted or shed form that is found in the supernatant of isolates that express high levels of the protease. Our results show that in exponential phase a considerable fraction of the protease is securely anchored to the bacterial surface, can be recognized by specific antibodies, and retains the ability to cleave chemokines. SpyCEP harbors a typical cell wall attachment motif, and surface localization through covalent attachment by the housekeeping sortase A would be expected. On the contrary, a considerable amount of the protease is consistently found in the supernatant of broth cultures during stationary phase. Since the protease undergoes autocatalytic processing, it was possible that shedding may depend, directly or indirectly, upon this activity. However, our data showed that shedding is independent of SpyCEP autoproteolytic activity. We speculate that shedding is related to *spyCEP* overexpression in the 3348 strain, and possibly contributes to the CovRS^−^ hypervirulent phenotype [22].

Previous work has suggested that mutants lacking *spyCEP* are less virulent in animal models, although there are conflicting results [4], [7]–[11]. *S. pyogenes* is an obligate human pathogen, and animal models of *S. pyogenes* infection are challenging to implement. We were interested in developing a model suitable for studying the interplay between bacteria and host, specifically the contribution of SpyCEP to host cell recruitment to the site of infection. Subcutaneous teflon chamber implants have been used with mice to study local responses to infection and host cell recruitment, but this model requires a surgical procedure and a post-surgical recovery period [30]. The air pouch route of infection has been used for *S. pyogenes* as a tissue damage model (ulceration and/or histopathology) or a lethal challenge model [31]. A similar model has been used by immunologists for several decades to evaluate immunomodulatory drugs and acute inflammation, where the main readout is inflammatory parameters [32]. In a typical setup, an air pouch is inflated in the mouse by repeated subcutaneous injections of air over time, an irritant is injected together with a putative modulator or control, and a lavage is then collected and analyzed [33], [34]. We adapted this protocol and replaced the irritant with a bacterial infection. In this work we show that the chemokines KC, LIX and MIP-2 are highly susceptible to SpyCEP action *in vivo*. In the strain lacking SpyCEP, levels of the above chemokines were 10–1,000 fold higher compared to the wild type strain. These three chemokines all promote PMN recruitment. In fact, mice infected with the mutant strain repeatedly showed higher levels of neutrophils and monocytes compared to w.t. infections, although the difference was not statistically significant (*P* = 0.1). We speculate that even the lower levels of chemoattractants in mice infected with the w.t. may be sufficient for near maximal recruitment to the site of infection. While it is clear from our work and that of others that cleaved IL-8 and KC fail to recruit PMN, we cannot rule out that LIX or MIP-2 might retain part or all of their chemoattractant properties even when cleaved, or, that other chemoattractants are involved (e.g. C5a). As for bacterial survival, at 4 h post-infection, mice infected with 3348Δ*spyCEP* showed bacterial growth retardation compared to mice infected with the 3348 w.t. strain, consistent with an elevated number of professional phagocytes in the former case.

Some general observations from our model of infection can also be made. One of the most prominent chemokines in the air pouch lavage was IL-6, a proinflammatory chemokine with prognostic value for severity of infection [7], [35], [36]. In our case, the high levels of IL-6 suggest that substantial general inflammation was taking place *in situ*, while we did not observe any difference between w.t. and mutant. However, the levels of IL-6 were in the uppermost end of the range covered by our assay, where values are distinctly less precise. A rather surprising finding was the high number of eosinophils recruited to the site. Eosinophils are not known to have any role in host defense against *S. pyogenes* infection, and neither are they particularly numerous among circulating leukocytes. Eosinophils are recruited during mycobacterial infection, particularly in lung granulomas, and it has been suggested that they play a direct role in TLR2-mediated innate immunity against mycobacteria [37]. We speculate that eosinophils may also have a role in *S. pyogenes* localized infection.

More extensive studies, including different infection doses, histopathology, the study of systemic dissemination from the site etc., could eventually clarify the extent to which SpyCEP affects overall outcome of infection in our model. We believe that this model of local infection holds great promise for the study of host-pathogen interactions, as it permits quantification of many interrelated infection parameters. Compared to traditional lethal challenges the model is fast, relatively harmless to the animals, and does not require complicated animal manipulation techniques. It should be applicable also to study virulence factors of other bacterial species, and we are currently investigating whether it can also serve as a model to investigate protective immunity.

We have shown that SpyCEP can cleave chemokines not only in its shed form, but also on the bacterial surface. Moreover, we have shown that specific antibodies to SpyCEP can counteract the inhibitory effects on PMN recruitment *in vitro*. Such antibodies can also interact with SpyCEP at the bacterial surface. Importantly, SpyCEP acts on chemokines *in situ*, and absence of the protein puts bacteria at a relative disadvantage. In the context of a vaccine against *S. pyogenes* infection, the inclusion of SpyCEP as a component may exert a dual effect – the promotion of opsonophagocytosis and a relative reduction of virulence

## Supporting Information

Figure S1 **Growth curves for 3348, 3348Δ** ***spyCEP*** **, 3348** ***spyCEP*** *, SF370, SF370Δ ***spyCEP*** **and SF370** spyCEP* **strains in THY.** Bacteria were grown until early exponential phase (OD = 0.2) and then diluted 1∶50 in fresh THY medium. The growth was followed by recording OD_600_ at 30 min intervals. (**A**) 3348 and mutants. (**B**) SF370 and mutants.
(TIF)

Figure S2 **SF370 infection in a murine air pouch model.** After air pouch inflation, CD1 female mice were infected with 1×10^8^ CFU of SF370 (n = 6, two mice were excluded due to in-fighting prior to infection), SF370Δ*spyCEP* (n = 8) or PBS (n = 3). 4 or 24 h post infection lavage material from each mouse was serially diluted and bacterial viable counts were performed. Multiplication factor (total CFU/inoculum CFU) in the lavage from individual mice is shown. Horizontal bars are geometric means.
(TIF)
