# Supplementary figures and images for: Streptococcus pyogenes SpyCEP Influences Host-Pathogen Interactions during Infection in a Murine Air Pouch Model
Source: PLoS One. 2012 Jul 27;7(7):e40411. doi: 10.1371/journal.pone.0040411 (PMC3407228; doi:10.1371/journal.pone.0040411)

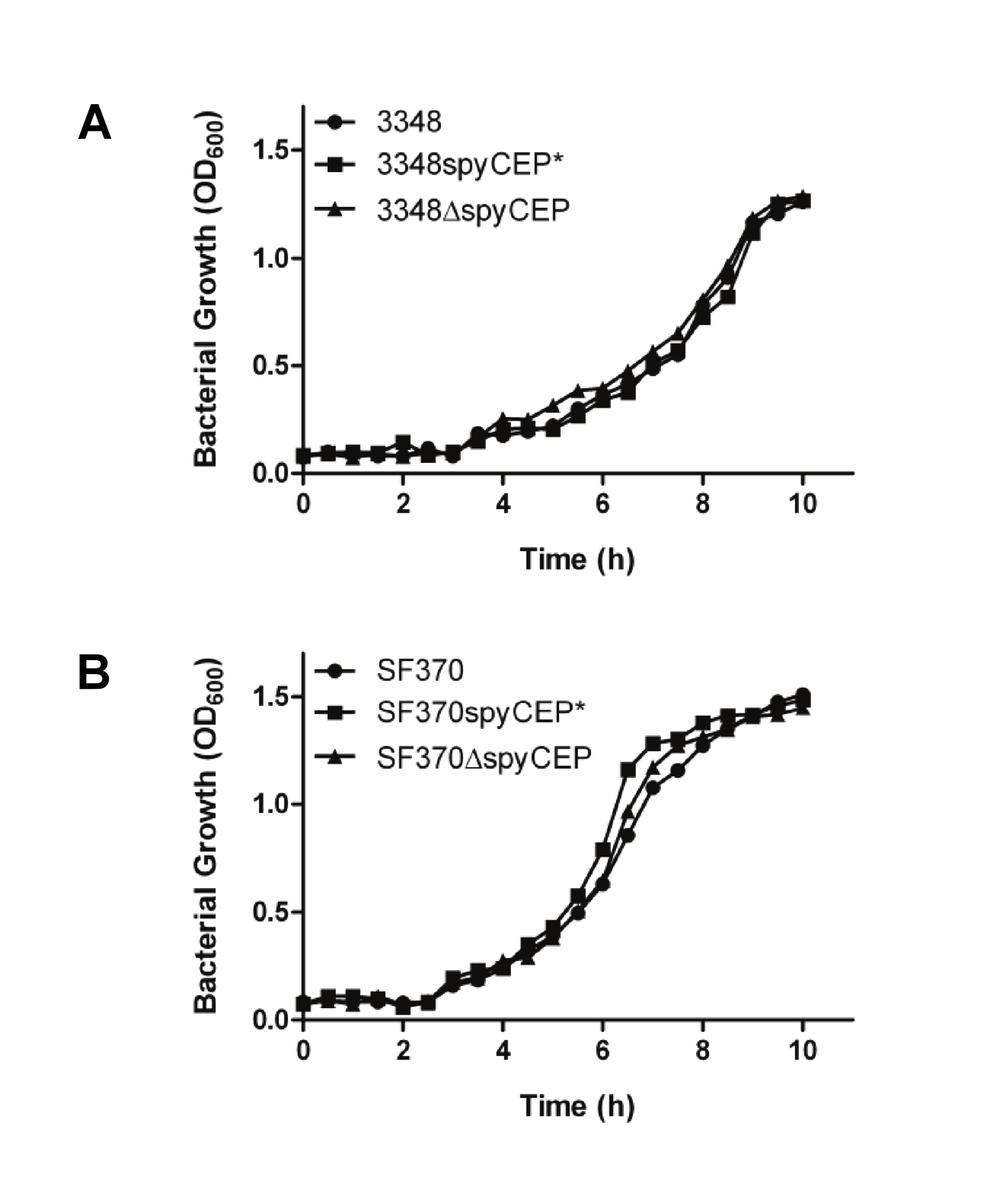

Supplement: Figure S1 — Growth curves for 3348, 3348Δ spyCEP , 3348 spyCEP *, SF370, SF370Δ spyCEP and SF370 spyCEP* strains in THY. Bacteria were grown until early exponential phase (OD = 0.2) and then diluted 1∶50 in fresh THY medium. The growth was followed by recording OD600 at 30 min intervals. (A) 3348 and mutants. (B) SF370 and mutants. (TIF) [file pone.0040411.s001.tif]

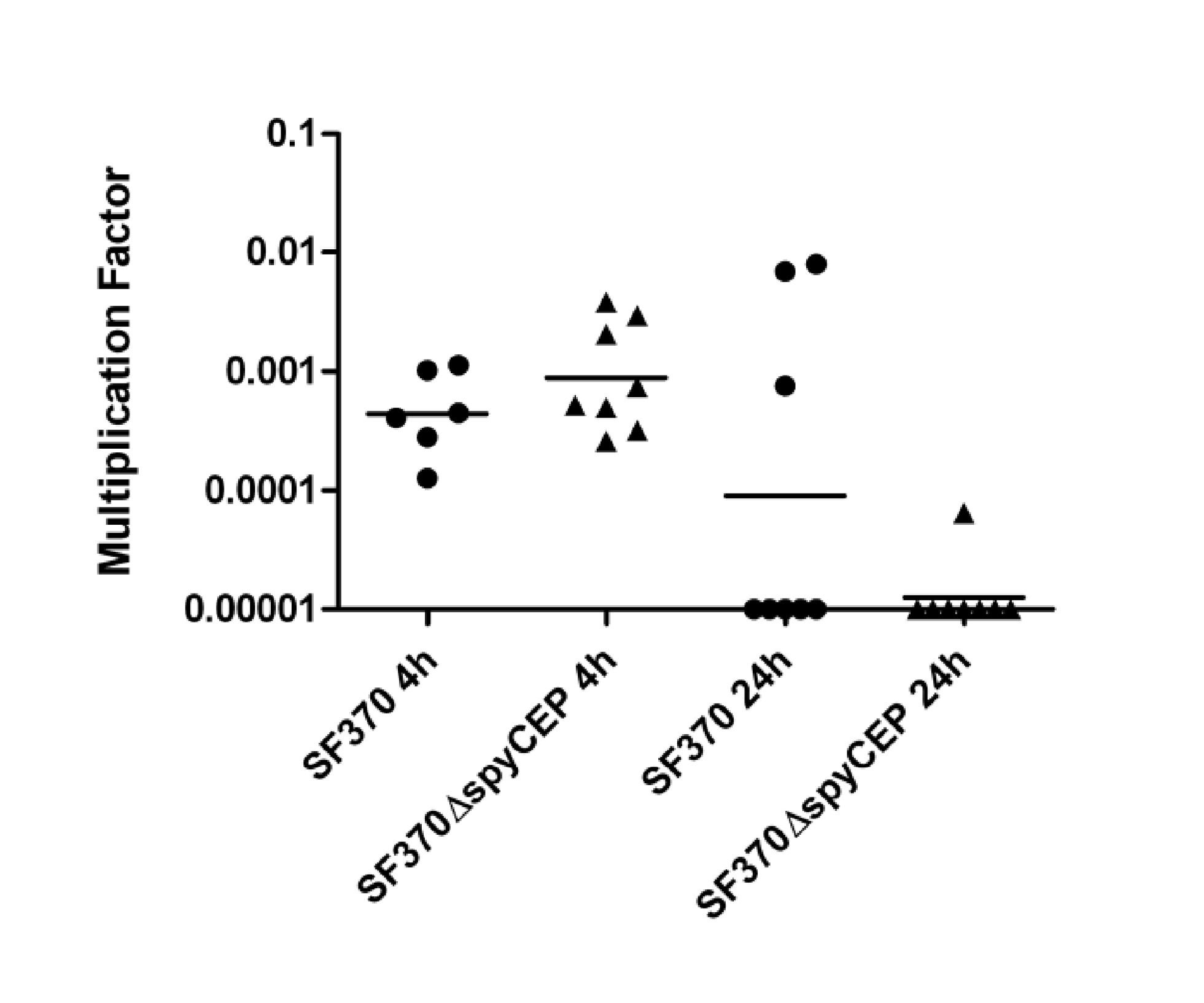

Supplement: Figure S2 — SF370 infection in a murine air pouch model. After air pouch inflation, CD1 female mice were infected with 1×10^8^ CFU of SF370 (n = 6, two mice were excluded due to in-fighting prior to infection), SF370ΔspyCEP (n = 8) or PBS (n = 3). 4 or 24 h post infection lavage material from each mouse was serially diluted and bacterial viable counts were performed. Multiplication factor (total CFU/inoculum CFU) in the lavage from individual mice is shown. Horizontal bars are geometric means. (TIF) [file pone.0040411.s002.tif]
